# Variation of 4 MV X-ray dose rate strongly impacts biological response both *in vitro* and *in vivo*

**DOI:** 10.1038/s41598-020-64067-4

**Published:** 2020-04-27

**Authors:** M. Ben Kacem, M. A. Benadjaoud, M. Dos Santos, F. Soysouvanh, V. Buard, G. Tarlet, B. Le Guen, A. François, O. Guipaud, F. Milliat, V. Paget

**Affiliations:** 1 Institute for Radiological Protection and Nuclear Safety (IRSN), Department of RAdiobiology and Regenerative Medicine (SERAMED), Laboratory of Medical Radiobiology (LRMed), Fontenay-aux-Roses, 92260 France; 2 Institute for Radiological Protection and Nuclear Safety (IRSN), Department of Radiobiology and Regenerative Medicine (SERAMED), Fontenay-aux-Roses, France; 3 Institute for Radiological Protection and Nuclear Safety (IRSN), Department of Radiobiology and Regenerative Medicine (SERAMED), Laboratory of Radiobiology of Accidental Exposures (LRAcc), Fontenay-aux-Roses, France; 4 Electricité de France, Cap Ampère, Saint-Denis, France

**Keywords:** Senescence, Cell-cycle exit

## Abstract

Whereas an RBE > 1 is described for very low-energy X-ray beams (in the range of 25–50 kV), there is a consensus that the RBE of X-rays (from 0.1 to 3 MeV) is equal to 1, whatever the energy or dose rate of the beam. Comparisons of X-ray beam dose rates are scarce even though these beams are widely used in medical diagnosis or radiotherapy. By using two dose rates (0.63 and 2.5 Gy.min^−1^) of high-energy X-rays on normal endothelial cells (HUVECs), we have studied the clonogenic assay, but also viability/mortality, cell cycle analysis and measured cellular senescence by flow cytometry, and have performed gene analysis on custom arrays. In order to consolidate these data, we performed localized irradiation of exteriorized small intestine at 0.63 and 2.5 Gy.min^−1^. Interestingly, *in vivo* validation has shown a significantly higher loss of weight at the higher dose when irradiating to 19 Gy a small fragment of exteriorized small intestine of C57Bl6J mice. Nevertheless, no significant differences were observed in lesioned scores between the two dose rates, while bordering epithelium staining indicated twofold greater severe damage at 2.5 Gy.min^−1^ compared to 0.63 Gy.min^−1^ at one week post-irradiation. Taken together, these experiments systematically show that the relative biological effectiveness of photons is different from 1 when varying the dose rate of high-energy X-rays. Moreover, these results strongly suggest that, in support of clonogenic assay, multiparametric analysis should be considered to provide an accurate evaluation of the outcome of irradiated cells.

## Introduction

Relative biological effectiveness (RBE) is the ratio of the dose of one kind of ionizing radiation relative to another to produce the same biological effect. Several studies have focused on dose rate effects, but they were mainly performed at low dose rates and by using ^137^Cs^1,2^ or ^60^Co^3,4^ sources. A few studies have directly compared the *in vitro* effects of different dose rates of X-rays on cancer cells, but not on normal human cells^5–9^. It is also known that RBE increases as LET increases up to 100 KeV.µm^−1^, above which RBE decreases because of cellular overkill^10^. Moreover, RBE for protons is also described as endpoint-dependent^11^, while there is a consensus that the RBE of X-rays (photons; energy from 0.1 to 3 MeV) is equal to 1, whatever the energy or dose rate of the beam^12^. Importantly, higher RBE is described for very low-energy X-ray beams (in the range of 25–50 kV)^13–16^. Nevertheless, modern radiotherapy uses medical devices (mostly 6–10 MV) able to deliver doses up to 20 Gy.min^−1^, assuming that the RBE of the X-ray beam remains equal to 1 whatever the energy and/or dose rate.

To verify this, and build a proof of concept both *in vitro* and *in vivo*, we set as our reference a beam of high energy X-rays (4 MV) at 0.63 Gy.min^−1^ on a Linear Accelerator (LINAC) Elekta Synergy Platform. Independently of the radiation type and delivery technique, several dosimetric studies of water phantoms or of real treatment planning conditions^17–19^ show that the lateral dose generally drops to 10% within 1 cm of the edge field. Therefore, by targeting a tumor at a dose rate up to 6 Gy.min^−1^, 0.63 Gy.min^−1^ appears to be a representative dose rate exposure for organs at risk quite close to the planning treatment volume. Our beam of interest was set at 2.5 Gy.min^−1^ (4-fold), in order to stay at strictly the same energy (4 MV). Importantly, both of these dose rates are in the range of the beams used in conventional radiotherapy^20^.

Human Umbilical Vein Endothelial Cells (HUVECs) were irradiated *in vitro* in clonal conditions (clonogenic assay) and at confluence for all other assays (viability/mortality, cell cycle, senescence and gene analysis on custom arrays). The overall results clearly indicate that the higher dose rate (2.5 Gy.min^−1^) of high energy X-rays significantly induced more adverse effects in HUVECs than a 4-fold lower dose rate (0.63 Gy.min^−1^). Furthermore, *in vivo* experiments also showed that an increase in dose rate induced a significantly greater loss of weight when irradiating at 19 Gy a small fragment of exteriorized small intestine of C57Bl6J mice. Moreover, bordering epithelium staining of the lesion showed that severe injury was significantly greater at 2.5 Gy.min^−1^ than at 0.63 Gy.min^−1^. Our findings clearly show that the RBE of X-rays (energy from 0.1 to 3 MeV) is not equal to 1 when changing the dose rate, both *in vitro* and *in vivo*.

## Results

### Clonogenic assay

**Figure 1:**
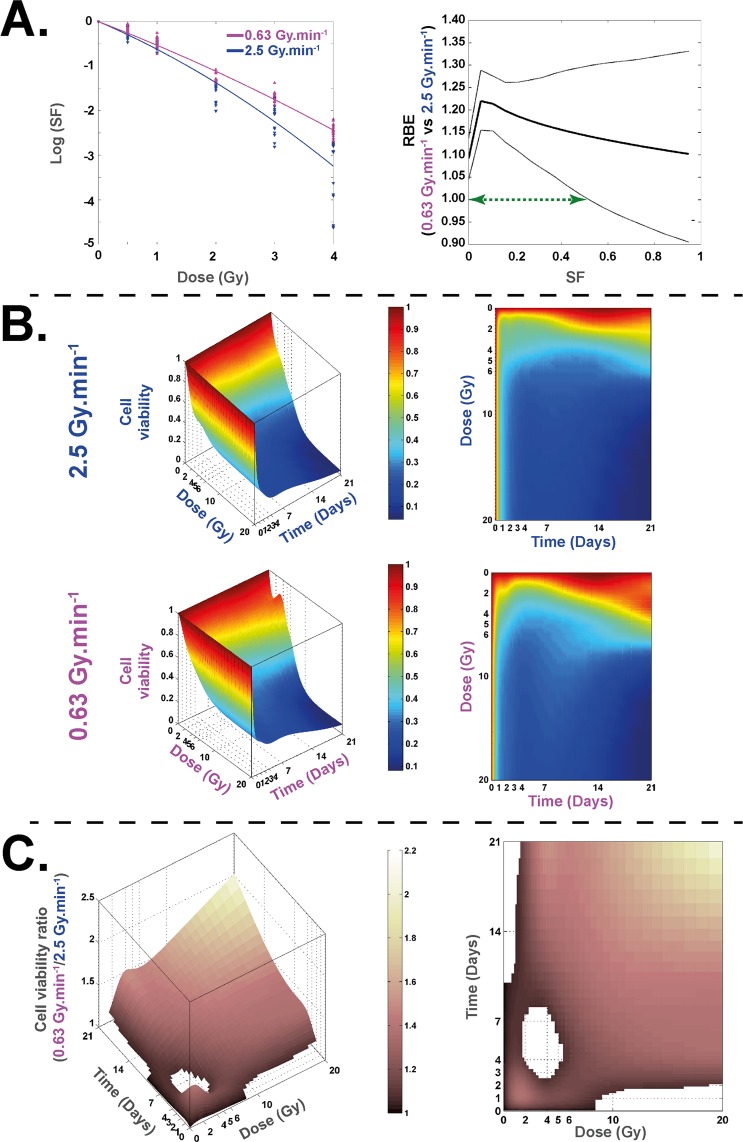
Clonogenic assay and cell survival at 0.63 and 2.5 Gy.min^−1^. (**A**) Left panel: Survival fraction (SF) of HUVECs irradiated at 0.63 Gy.min^−1^ (pink curve) and 4 MV (blue curve). Right panel: the associated RBE curve is defined as a ratio of doses for a given SF (thick black line) and its associated bootstrap confidence intervals (two fine black lines). The green arrow represents the range of SF wherein the value of RBE is significantly different from 1. (**B**) Representations of 3D and 2D curves of cell survival measures with trypan blue counting method for 0.63 and 2.5 Gy.min . (**C**) Representations of 3D and 2D curves of cell viability ratio [0.63/2.5 Gy.min^−1^].

For both irradiations, the survival fraction decreased in a dose-dependent manner (Fig. 1A). A significant difference between the two beams of irradiation throughout the 1 to 4 Gy dose range was found, and RBE values were statistically significantly different from 1 between the two dose rates for SF ≤ 0.55 (Supplementary Table S1).

### Cell viability

Cell viability counting using the trypan blue method (Fig. 1B), shows higher cell viability after irradiation at 0.63 Gy.min^−1^ compared to 2.5 Gy.min^−1^. A statistical representation of (0.63 Gy.min^−1^)/(2.5 Gy.min^−1^) cell viability ratios and associated statistical results is shown in Fig. 1C, where cell viability was significantly higher at the lowest dose rate, excepted for a time/dose range visualized by a “white hole” on the surface where no significant difference was found.

### Cell cycle

**Figure 2:**
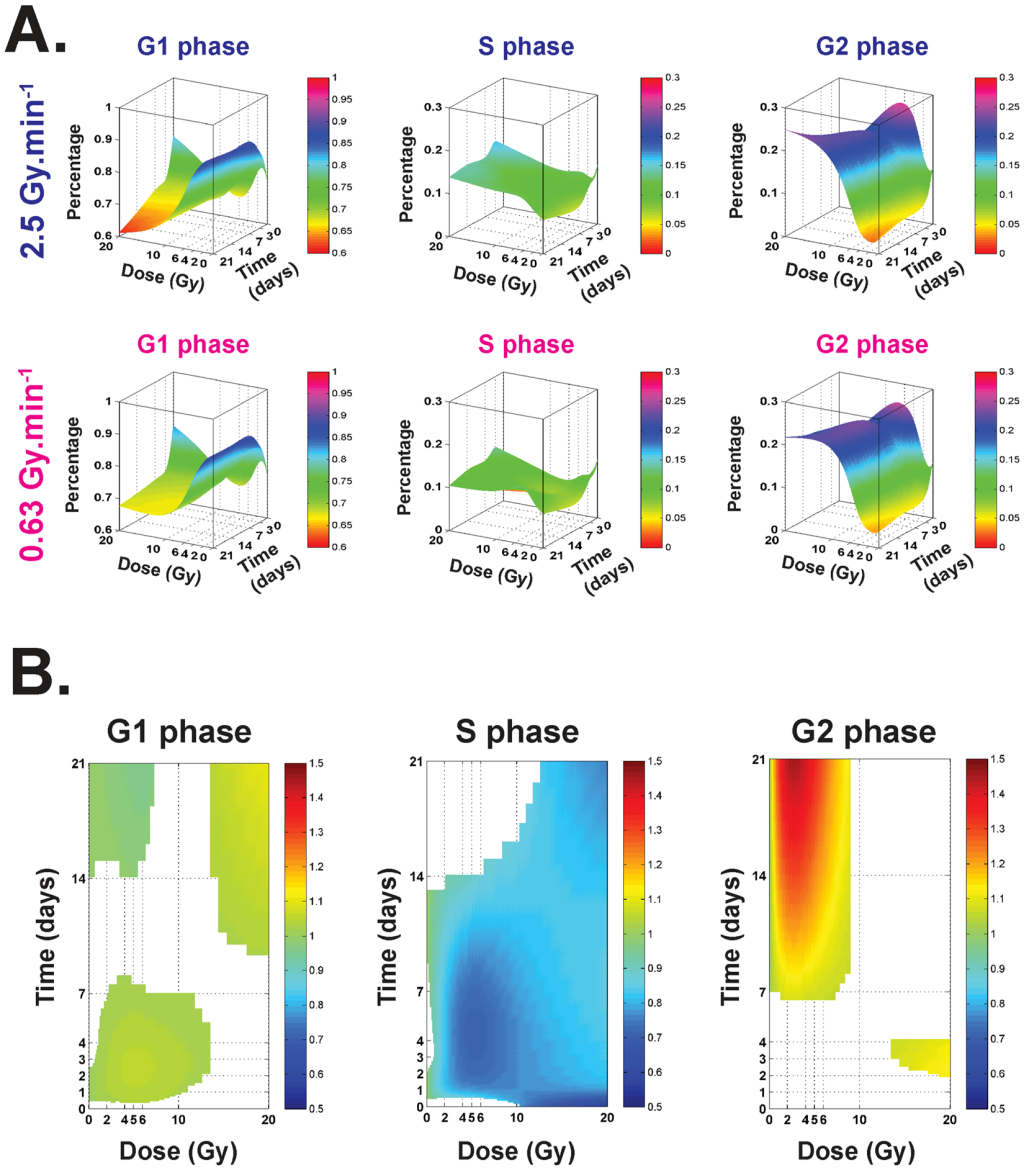
Cell cycle proportions at 0.63 and 2.5 Gy.min^−1^. (**A**) Modeling of cell cycle proportions (G1, S and G2) according to the dose and time (surfaces represent the mean of at least three independent experiments). (**B**) Representations of 2D curves of the cell cycle ratio [0.63/2.5 Gy.min^−1^] for the phase (G1 (left), S (middle) and G2 (right). Colored surfaces represent statistically significant differences between the two dose rates. Colors indicate the value of the ratio.

Modeling of cell cycle proportions (G1, S and G2) according to the dose and time were modeled for each dose rate (Fig. 2A). Then, when considering the cell cycle ratio [0.63/2.5 Gy.min^−1^] (Fig. 2B) for each phase (G1 (left), S (middle) and G2 (right)), statistically significant differences are represented by colored surfaces, the color indicating the corresponding value of the ratio (Fig. 2B). This approach allowed us to determine ranges of doses and/or scale of time, where dose rate significantly impacts the cell cycle.

### Senescence

**Figure 3:**
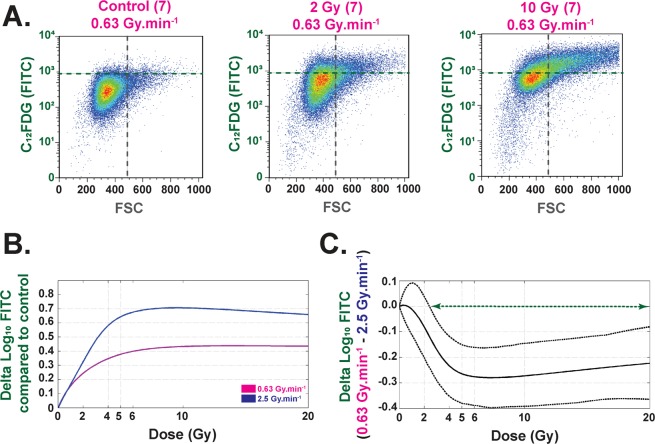
Senescence (C FDG) (Flow Cytometry) analysis. (**A**) Example of flow cytometry measurements obtained at D7 for control, 2 and 10 Gy irradiations at 4 MV and 0.63 Gy.min^−1^. Each bi-parametric representation (Size (FSC)/C FDG (FITC)) represents one independent experiment for at least 5x10 living cells. (**B**) Each curve (purple and blue for 0.63 and 2.5 Gy.min, respectively) represents the delta of Log FITC compared to control conditions. Each curve represents the mean of three independent experiments based on at least 5x10 living cells for each experiment. (**C**) The curve represents the delta log FITC (0.63-2.5 Gy.min^−1^) as a function of the dose. The green arrow represents the range of dose where this delta is significant between the two dose rates (from 2.2 to 20 Gy).

Using the C_12_FDG fluorescent substrate and flow cytometry assays, our experiments demonstrate that the number of senescent cells and the size of the cells increase with dose for both dose rates (Fig. 3A,B and Supplementary Fig. S2). Nevertheless, our results systematically show, for each dose from 2.2 to 20 Gy, a significant difference between 0.63 and 2.5 Gy.min^−1^ irradiations at 4 MV (Fig. 3B, right panel). Senescence RBE values were calculated and are reported in Supplementary Table S2.

### Senescence-associated gene expression clustering

**Figure 4:**
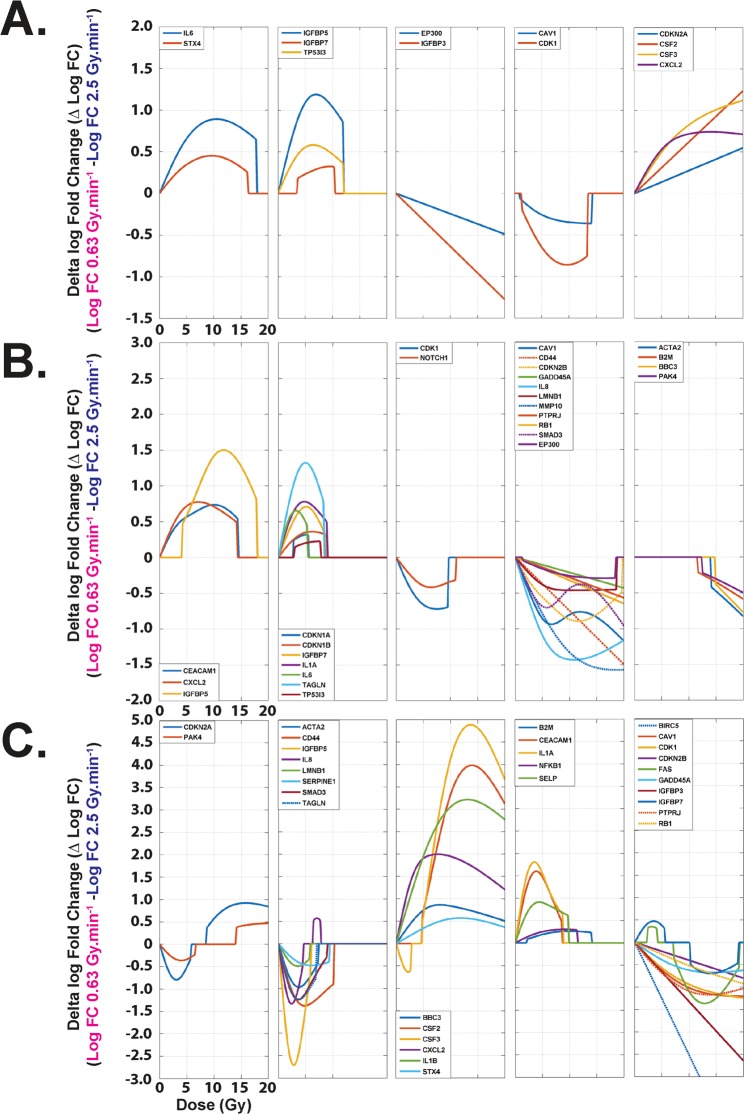
RT-qPCR gene expression clustering. (**A**) Gene clusters at D3 post-irradiation, each curve representing the delta of fold changes for one gene (0.63 − 2.5 Gy.min^−1^). Genes were grouped in five clusters according to their expression tendencies as a function of the dose. Only the significantly differentially expressed genes in the delta fold change (13 among the 44 measured) are represented. (**B**) Gene clusters at D7 post-irradiation. Genes were grouped in five clusters according to their expression tendencies as a function of the dose. Only the significantly differentially expressed genes in the Delta fold change (27 among the 44 measured) are represented. (**C**) Gene clusters at D21 post-irradiation. Genes were grouped in five clusters according to their expression tendencies as a function of the dose. Only the significantly differentially expressed genes in the delta fold change (31 among the 44 measured) are represented.

An example of gene expression unsupervised hierarchic clustering was established based on data obtained from RT-qPCR gene expression measurements of 44 senescence-associated genes using a customized Taqman Low-Density Assay (TLDA) (Supplementary Fig. S4). For each end-point (Day 3, 7 and 21), clusters were defined by tendencies of delta fold change according to the dose. At day 3 post-irradiation, five clusters of expression were obtained considering the 13 statistically differentially expressed genes according to the dose rate (Fig. 4A). At day 7 post-irradiation, 27 genes were significantly differentially expressed (Fig. 4B). At day 21 post-irradiation, significantly differentially expressions according to the dose rate were found for 31 genes hosted on 5 different clusters (Fig. 4C).

### *In vivo* weight follow-up

**Figure 5:**
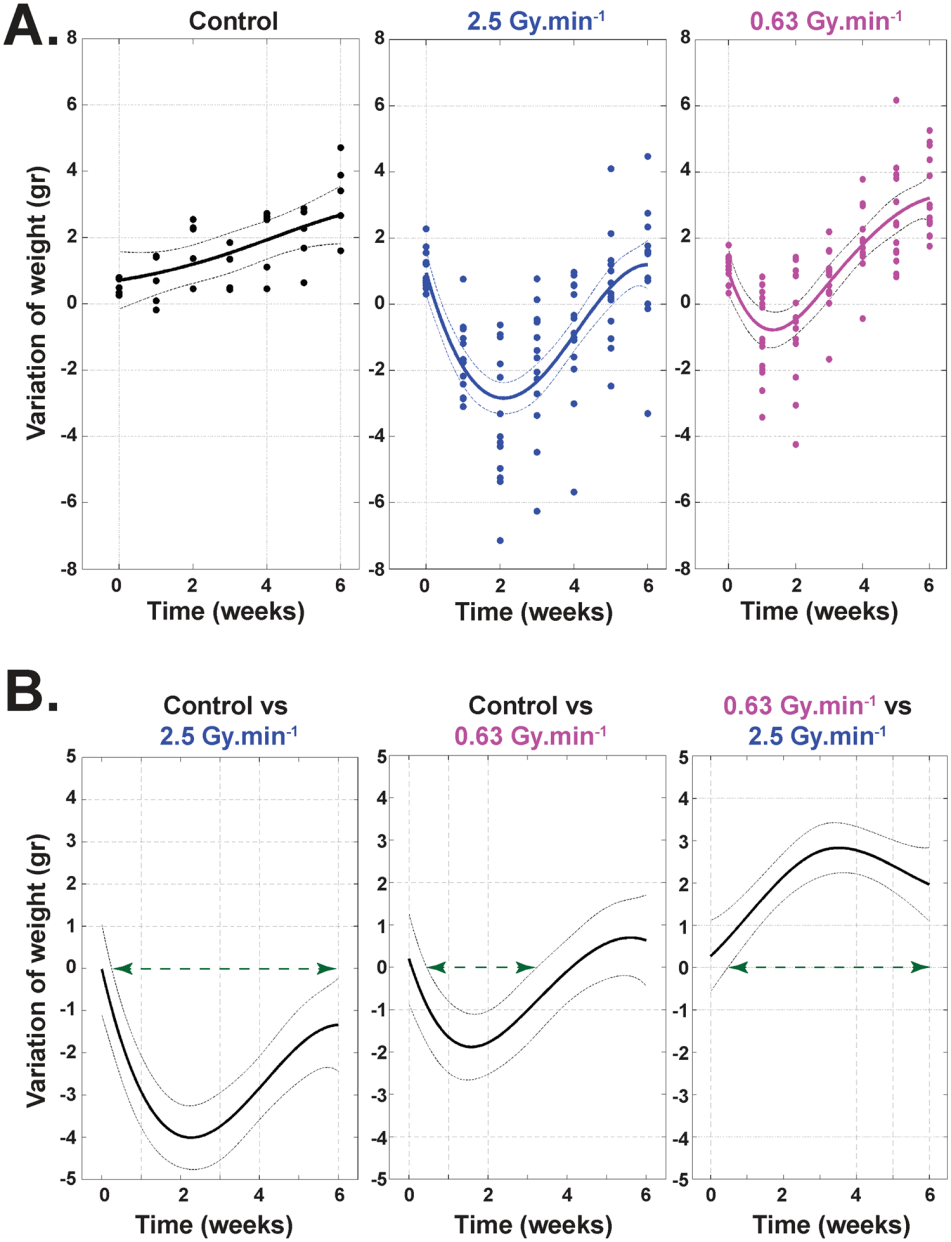
Weight follow-up of animals. (**A**) Follow-up of the animals’ weights from 0 to 6 weeks after irradiation, with sham-irradiated controls (left panel, n =5 animals), after 19 Gy irradiation at 2.5 Gy.min^−1^ (middle panel, n = 12 animals), and after 19 Gy irradiation at 0.63 Gy.min^−1^ (right panel, n = 12 animals). (B) Statistical representation of the loss of weight, control versus 2.5 Gy.min^−1^ irradiation (left panel), control versus 0.63 Gy.min^−1^ irradiation (middle panel) and 0.63 versus 2.5 Gy.min^−1^ irradiation (right panel). For each panel, the green arrow represents the range of doses for which there is a statistically significant difference between the two considered conditions.

Weight follow-up from T_0_ to 6 weeks after irradiation shows a greater loss of weight for the 2.5 Gy.min^−1^ irradiation compared to the 0.63 Gy.min^−1^ (Fig. 5). Moreover, while statistically significant loss of weight was found from 0.5 to 6 weeks after irradiation for the 2.5 Gy.min^−1^ (Fig. 5B, left panel), the loss of weight was only statistically significant from 0.5 to 3 weeks for the 0.63 Gy.min^−1^ irradiation compared to the control mice (Fig. 5B, middle panel). Finally, when comparing results for both dose rates (0.63 versus 2.5 Gy.min^−1^), statistically significantly greater weight was found for the lowest dose rate from 0.5 to 6 weeks post-irradiation (Fig. 5B, right panel).

### *In vivo* radiation injury scoring

**Figure 6:**
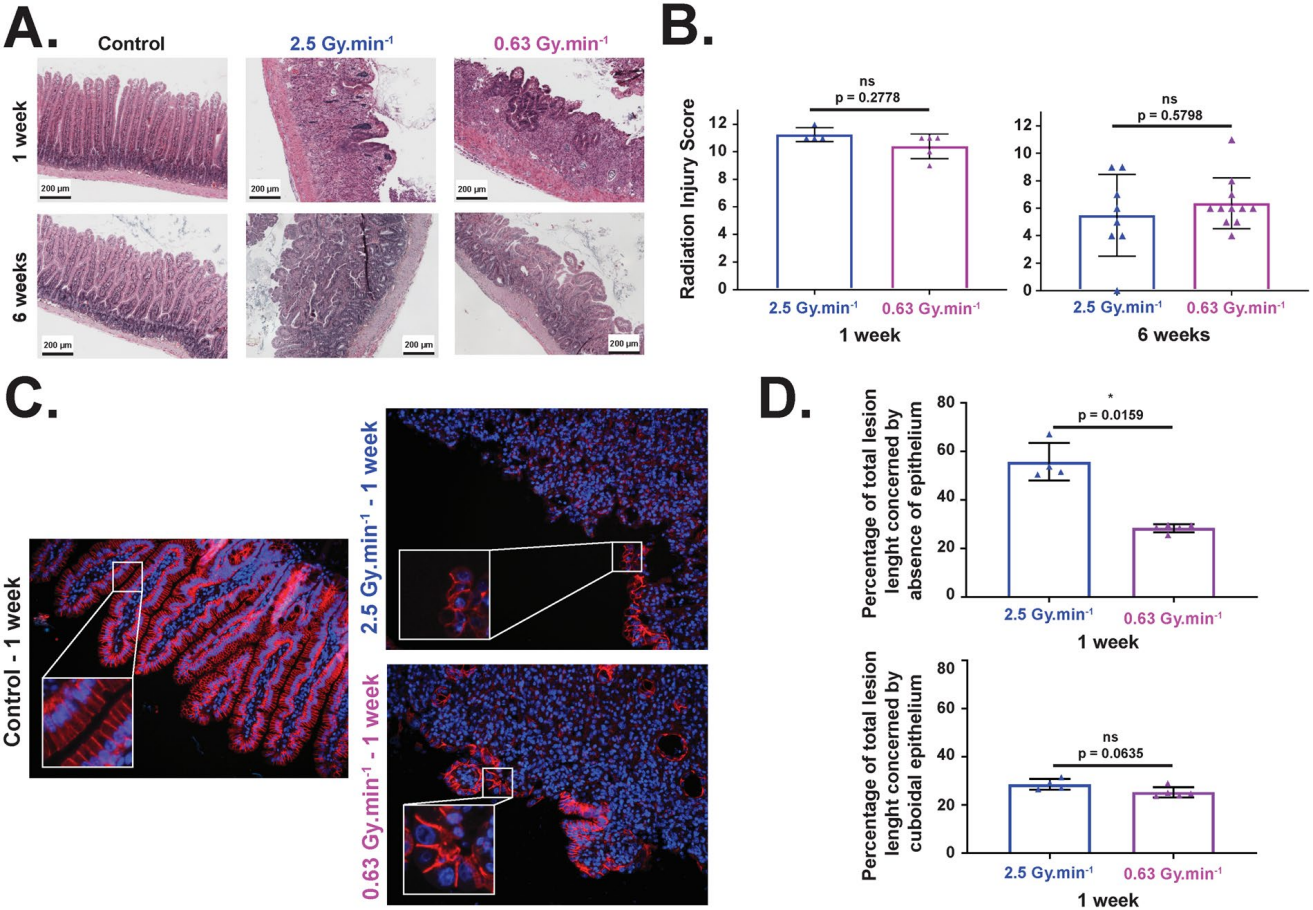
Histological analysis and lesion scorings after 19 Gy localized intestinal radiation exposure. (**A**) Representative microscopic alterations obtained in 0.63 and 2.5 Gy.min^−1^ mice 1 and 6 weeks after irradiation. Slides were stained with hematoxylin-eosin-saffron. Scale bar = 200 μm. (**B**) A semi-quantitative radiation injury score was assigned to irradiated tissues from both beams 1 and 6 weeks after irradiation (n = 6 animals per group for 1 week end-point, n = 12 animals per group for 6 week end-point, adherences were removed from the figure). (**C**) Examples of p120 catenin staining performed at 1 week on small intestine section of non-irradiated mice (left panel), and mice irradiated at 2.5 Gy.min^−1^ (right upper panel) and 0.63 Gy.min^−1^ (right bottom panel) (original magnification x200). (**D**) Percentages of bordering epithelium with severe damage (upper panel) and cuboidal cells (bottom panel) (^∗^, p < 0.05).

Lesion scoring was performed according to^21^ on sections stained with HES (Fig. 6A), taking into account 8 parameters included in the Radiation Injury Score (RIS). Samples showing intestinal adherences were removed from the group to avoid misinterpretation of the data. Using this scoring, we found no statistically significant difference in the injury score between the two dose rates for the two end-points (1 and 6 weeks post-irradiation) (Fig. 6B). Mucosal damage was evaluated by measuring the percentage of total lesions affecting i) healthy bordering epithelium which corresponds to well-oriented/elongated columnar cells with basal nucleus (Fig. 6C, left panel, zoom in control conditions), and ii) atypical epithelium corresponding to cuboidal epithelial cells with centered nucleus (Fig. 6C, right panels, zoom in both irradiated conditions). Using these parameters, irradiation at 2.5 Gy.min^−1^ induced a significantly higher percentage of severe damage to the bordering epithelium compared to 0.63 Gy.min^−1^ (Fig. 6D, upper panel) at 1 week post-irradiation. On the other hand, no statistically significant difference (even very close to a p < 0.05) was observed between the two beams concerning the percentage of cuboidal epithelium (Fig. 6D, bottom panel). Severe damage RBE was estimated (ratio of severe damage percentages for a given dose) and is reported in Supplementary Table S3.

## Discussion

The present study assessed *in vitro* cellular outcome up to 21 days after 4 MV X-ray irradiation, using two dose rates (0.63 and 2.5 Gy.min^−1^), and covering a wide range of doses (0, 2, 4, 5, 6, 10 and 20 Gy). HUVECs were chosen as a biological model due to their capability to form clones in dishes^22^ and because vascular injury is among the most common effects of radiotherapy of normal tissues and tumors^23,24^. Furthermore, radiotherapy is known to induce vascular permeability, characterized by an increase of albumin leakage after single-dose irradiation from 2 to 30 Gy or more, thus fully justifying the biological relevance of our range of doses^25,26^. Finally, this study uses human primary cells, while the literature data concerns several human cancer cells^6,27–30^, Chinese hamster cells^6,28,29,31^ and rat cells^27^.

Our statistical analysis of the clonogenic assay, which is regarded as the reference in assessment of RBE^32,33^, shows that the two modalities of irradiation have an RBE (0.63/2.5) significantly different from 1 for an SF ≤ 0.55 and that peaks at 1.29 (Fig. 1A and Supplementary Table S1). To verify these results, we expanded the study to other assays such as cell viability counting. We calculated a relative dose effect showing significantly greater cell death at the higher dose rate, thus corroborating our results in clonal conditions. As several published studies focused on dose rate effects are essentially based on the clonogenic assay^6,30,31^ or DNA damage^1–4^, we decided to investigate further the phenotype of surviving cells in both conditions. Thus, we evaluated and modeled the impact of the dose rate on the cell cycle between the two beams, ensuring beforehand that before irradiation cells were mainly in G1 phase (Supplementary Fig. S2, around 70–80%), as it is well known that cell cycle phase can strongly affect the results after irradiation^34^. Furthermore, previous studies on cancer cell lines found no significant differences in cell cycle phases when dose rate was changed^28^. Interestingly, statistical analysis of our results on human normal endothelial cells revealed significant differences between the two dose rates, such as fewer cells in phase S at 0.63 Gy.min^−1^ compared to 2.5 Gy.min^−1^. As the ratio of this proportion is below 1, this could indicate (i) more proliferation at 2.5 Gy.min^−1^ than at 0.63 Gy.min^−1^, or (ii) the opposite, cell cycle blockage in S phase significantly higher at 2.5 Gy.min^−1^ than at 0.63 Gy.min^−1^ (Fig. 2). In point of fact, radiation-induced senescence is now well described and is characterized by increases in cell size and β-galactosidase activity^35^. Interestingly, senescent cells are blocked in the cell cycle^36^, but remain metabolically active. Furthermore, once engaged in the senescence process, cells fail to initiate DNA replication^36,37^. To verify radiation-induced senescence *in vitro* in HUVECs, we performed staining with C_12_FDG instead of the widely used biomarker X-GAL^38,39^. C_12_FDG was chosen so as to use flow cytometry, which is very sensitive for a very large number of events depicting a representative response of the whole cell monolayer^38^. As reported by Debacq-Chainiaux *et al*.^38^, we also used bafilomycin A1 pre-treatment in our experiments, so as to be more specific to β-galactosidase activity linked with stress-induced senescence. Interestingly, we found significantly more senescent cells at 2.5 Gy.min^−1^ compared to 0.63 Gy.min^−1^ for doses above 2.2 Gy (Fig. 3), thus corroborating the significantly higher proportion of cells in S phase at 2.5 Gy.min^−1^ compared to 0.63 Gy.min^−1^. The differential profile of radiation-induced senescence in connection with the dose rate was also confirmed by custom TLDA of 44 genes reported to be involved in the senescence process and the senescence-associated secretory phenotype (SASP)^35,36,40^. Basic analysis by gene clustering (“heat map”) a dose rate effect for the 10 and 20 Gy conditions at day 21 post-irradiation (Supplementary Fig. S4). When analyzing gene deregulation in more detail, our statistical analysis sorted 13, 27 and 31 genes statistically differentially expressed according to the dose rate, at days 3, 7 and 21 post-irradiation, respectively (Fig. 4A–C, respectively). From a general standpoint, this increase of deregulated genes over time clearly indicates a dose rate effect. Moreover, if we focus more precisely on key genes of the SASP, such as IL8 and MMP10, we show that their fold changes were statistically significantly greater at day 7 for the dose rate at 2.5 Gy.min^−1^ compared to 0.63 Gy.min^−1^ (Fig. 4B), thus fully corroborating the higher level of senescent cells detected by flow cytometry when increasing the dose rate.

Finally, in order to verify our *in vitro* data, we performed *in vivo* experiments. To do so, we focused on a mouse model of radiation-induced enteropathy^41,42^. Weight loss of animals following irradiation is an important parameter in evaluating the severity of radiation-induced damage^43–46^. Modeling of weight measurements established a significant difference between the two dose rates from 0.5 to 6 weeks after irradiation, this loss of weight being systematically greater for the higher dose rate. Unfortunately, injury score^21^ measured on histological tissue sections did not show significant differences between the two dose rates, at 1 or 6 weeks after irradiation. Nevertheless, as this injury score integrates several parameters^47,48^, p120 catenin staining was performed to further investigate and characterize the lesion as previously done in our laboratory^48^. Interestingly, we found, one week post-irradiation, that severe damage on the bordering epithelium at 2.5 Gy.min^−1^ (Fig. 6) was twice that at 0.63 Gy.min^−1^. At the same time, one week post-irradiation corresponds to the climax of the decrease for 0.63 Gy.min^−1^, this climax being reached 2 weeks post-irradiation for 2.5 Gy.min^−1^ and marked by a significantly greater loss of weight compared to 0.63 Gy.min^−1^ (Fig. 5). All together, these two criteria (weight and bordering epithelium) fully corroborate and validate *in vivo* that an increase in dose rate induces more deleterious effects when using a mouse model of radiation-induced enteropathy.

Modern radiotherapy can use modifications of the energy electron beam, such as flattening filter free (FFF) to raise dose rates to around 20–24 Gy.min^−1^. Even though *in vitro* studies have reported a comparable cell survival between high dose rate FFF and conventional dose rate irradiation^8,31^, some authors have reported differences between FFF mode and a standard flattening beam, suggesting that the dose per pulse might be a crucial factor influencing cell survival^30^. Interestingly, we have recently demonstrated *in vitro* by using multiparametric radiological assays that a variation of X-ray energy strongly impacts RBE^49^. The present study shows, both *in vitro* on human normal cells and *in vivo* in mice, that the RBE of high-energy X-rays depends on the dose rate of the beam. Based on these findings, this clearly raises the question as to whether or not, when increasing the dose rate in radiotherapy, this could impact the cellular outcome for normal tissues and/or tumors. As there is a consensus that the RBE of X-rays (photons; energy from 0.1 to 3 MeV) is equal to 1, whatever the energy or dose rate of the beam, such methodology could also be helpful to compare RBE with dose rate modifications (in connection or not with energy modification), especially in the range of doses for which the clonogenic assay cannot be performed.

## Methods and Materials

### Irradiation on LINAC

Irradiation with high-energy X-rays was performed using an Elekta Synergy Platform (Elekta S.A.S. France, Boulogne, France) delivering 4 MV X-rays. Irradiations were performed under similar conditions: plate, cell culture medium for both dose rates (0.63 and 2.5 Gy.min^−1^) in air kerma free in air. The uncertainty in the dose rate measurement was about 7% for LINAC irradiations at k = 2.

### Cell culture

Human umbilical vein endothelial cells (HUVECs, C2519A) from Lonza were cultured in strictly the same conditions as widely described in the literature^49^.

### Clonogenic assay

Cells were irradiated on LINAC (0 (control), 0.5, 1, 2, 3 and 4 Gy) by following a protocol already described in the literature^22,49^.

### Viability/mortality (trypan blue)

The complete procedure of cell counting was previously described^49^.

### Cell cycle analysis

Cells were irradiated at 2, 4, 5, 6, 10 or 20 Gy or not irradiated (control). At T_0_, day 3, day 7 and day 21, cells were trypsinized. Each sample was centrifuged for 5 min at 200 g and the pellet was resuspended in 1 mL of [PBS (+MgCl_2_ + CaCl_2_) + 5% of FBS]. The resuspended cells were then fixed via the dropwise addition of 3 mL of 70% ethanol and the tubes were placed at – 20 °C at least overnight. At the end of the complete cycle, all samples were centrifuged to eliminate ethanol. Each pellet was rinsed twice with 5 mL of [PBS (+MgCl_2_ + CaCl_2_) + 5% of FBS]. Then the pellets were resuspended with 500 µL/tube of a mix containing [PBS (+MgCl_2_ + CaCl_2_) + 5% of FBS + propidium iodide (PI) (Sigma Aldrich, ref P4170) (at 25 µg/mL final) + RNAse A (50 µg/mL final)]. Finally, the tubes were placed at 4 °C overnight to stain the DNA. The next day, acquisition of the data was performed on a BD Facs CantoII using Diva. The first analysis was done using size/granulometry parameters. This first step allowed us to determine the gate (gate 1) where cells were recorded. Upon these gated events, the PI (Sigma Aldrich) signal was collected on the PE channel (Filter 585/42 nm) after air-cooled 488 nm solid state laser excitation (20 mW). As second analysis step the PE signal was plotted as PE-W versus PE-A in a dot plot graph, to discriminate doublets (eg, a G1 doublet from a G2/M single) or cellular aggregates^50^ and to record at least 5 × 10^4^ single events per replica (gate 2) upon which cell cycle analysis was performed using “Cell Cycle Tool” available on FlowJo 7.6.5 software.

### Senescence (C_12_FDG)

Cells were irradiated at 2, 4, 5, 6, 10 or 20 Gy or not irradiated (control). Seven days after irradiation, senescence experiments were performed by following Debacq-Chainiaux *et al*.^38^ using 1-hour pre-treatment with bafilomycin A1 (100 nM final), followed by addition of C_12_FDG (33 µM final) for 2 hours, as already described in one of our previous studies^49^. Supernatant was removed, monolayers were rinsed twice with PBS 1X (without Ca^2+^ and Mg^2+^), cells were trypsinized and centrifuged for 5 min at 200 g and the pellet was resuspended in 1 mL of PBS before acquisition on a FACS Canto II. To increase the robustness of the results, a cell viability reporter was added to each sample: To-Pro-3 before the acquisition of the data on a FACS Canto II (3-laser, 4-2-2 configuration) using FACS Diva software, 3 independent experiments were performed for each condition. Data analysis was performed post-acquisition using FlowJo 7.6.5 software (FlowJo LLC). A first analysis was done on size (FSC: forward scatter)/granulometry (SSC: side scatter) parameters, to collect cells (gate G1) and to remove fragmented cells and debris. Triton 0.06X final was instantly used as positive control (Supplementary Fig. S2A) to ensure good detection of dead cells. This first step allowed us to assess cell viability (on the APC channel (filters λ_em_: 660/20 nm) after 633 nm HeNe solid state (17 mW output) laser excitation) and to determine the gate (G2) where at least 5 × 10^4^ living cells per replica were recorded (Supplementary Fig. S2A, right panel). Then, upon this gated event (G2) the C_12_FDG signal was collected on the FITC channel (filters λ_em_: 530/30 nm) after air-cooled 488 nm solid state (20 mW output) laser excitation. DMSO (vector of C_12_FDG) was used as negative control to ensure good detection of C_12_FDG in controls (Supplementary Fig. S2B), but also for irradiated conditions (Supplementary Fig. S2D). Strictly the same acquisition parameters were applied to each condition containing C_12_FDG (Supplementary Fig. S2C,E,F for control, 2 Gy and 10 Gy, respectively) and bi-parametric analysis of cell size (FSC)/C_12_FDG (FITC) was performed (Supplementary Fig. S2G).

### RT-qPCR (custom TLDA)

Seven, 14 or 21 days after irradiation (at 0, 2, 4, 5, 6, 10 or 20 Gy), HUVECs were harvested with 600 µL per sample of mirVana miRNA Isolation Kit lysis buffer (ThermoFisher Scientific, AM1560). Total RNA was quantified on a ND-100 NanoDrop and samples were stored at −80 °C. Total RNA was diluted to 50 ng/µL (final concentration) and 500 ng was used to perform RT-PCR. cDNAs were loaded on customized TLDA. The PCR protocol was as follows: a preparation step (50 °C for two minutes followed by 10 min at 94.5 °C), then 40 cycles including denaturation (97 °C, 3 min), hybridization of primers and elongation (60 °C, 1 min). The Taqman Low Density Assay (TLDA) list of genes was previously described in the literature^49^. Analysis of data was performed using ExpressionSuite software (ThermoFisher Scientific), while representation and statistical analysis of the data were performed using DataAssist software (ThermoFisher Scientific).

### *In vivo* experiments

Animal experiments were performed in strict compliance with French and European guidelines and regulations on protection of animals used for scientific purposes (EC Directive 2010/63/EU and French Decree No. 2013–118). They were approved by the Ethics Committee on Animal Experiments of the IRSN, registered under number 81 and authorized by the French Ministry of Research under the reference APAFIS#16159-2018071812598405 v2 (internal project number P18-05).

Male C57BL/6JRj mice were from Janvier Labs (Saint Berthevin, France) and were 10 weeks of age upon arrival. Animals were housed in the IRSN animal facilities authorized by the French Ministry of Agriculture for performing experiments on rodents. After two weeks of acclimation, mice were anesthetized with isoflurane and, after abdominal laparotomy, a 3 cm-long intestinal segment (10 cm from the ileocecal valve) was exteriorized and exposed to a single dose of 19 Gy of high energy X-rays using the Elekta Synergy Platform (0.63 or 2.5 Gy.min^−1^). Sham-irradiation (Sham-IR) was performed by maintaining the intestinal segment exteriorized without radiation exposure. After radiation exposure or sham-irradiation, the exposed segment was returned to the abdominal cavity and peritoneum/abdominal muscles and skin were separately closed using with interrupted sutures^42^.

#### Tissue harvesting, histology, and immunohistology

At different times after radiation exposure, tissues were removed, fixed in 4% paraformaldehyde, and embedded in paraffin. Sections (5 μm) were stained with hematoxylin-eosin-saffron (HES).

#### Radiation injury score (RIS)

All the details regarding RIS variables and grades of radiation injury are widely described in the literature^47,48^.

#### p120 Catenin staining

p120-Catenin/F-actin co-immunostaining was performed as already described^48^. Cells were rinsed, fixed, permeabilized and primary rabbit monoclonal anti-p120 catenin antibody (Abcam, ab92514) (1/100 final) was added and incubated for 1 h at room temperature. Corresponding secondary antibody (Alexa Fluor 568-conjugated antibody, ThermoFisher Scientific, ref A11036) (1/250 final) was added for 1 h at room temperature. After rinsing, 4’,6-diamidino-2-phenylindole dihydrochloride (DAPI) (ThermoFisher Scientific, D1306) staining was performed for 10 min at room temperature at 0.2 µg/mL (final concentration). After rinsing, slides were mounted in Vectashield mounting medium without DAPI (Eurobio/Abcys).

### Statistical analysis

For a direct comparison of the two dose rate effects, we defined the RBE for each endpoint by considering 0.63 Gy.min^−1^ as a reference condition:

$$\frac{RB{E}_{refvs2.5Gy.mi{n}^{-1}}}{RB{E}_{refvs0.63Gy.mi{n}^{-1}}}=\frac{\frac{{X}_{ref}}{{X}_{2.5Gy.mi{n}^{-1}}}}{\frac{{X}_{ref}}{{X}_{0.63Gy.mi{n}^{-1}}}}=\frac{{X}_{0.63Gy.mi{n}^{-1}}}{{X}_{2.5Gy.mi{n}^{-1}}}=RB{E}_{0.63Gy.mi{n}^{-1}vs2.5Gy.mi{n}^{-1}}$$

#### Clonogenic assay

The number of scored colonies ${{\rm{y}}}_{{\rm{i}}}({\rm{d}})$ at each dose d and plate i, was modeled as a Bernoulli trial^51^:

$${{\rm{y}}}_{{\rm{i}}}({\rm{d}}) \sim {\mathfrak{B}}({{\rm{N}}}_{{\rm{i}}}({\rm{d}}),\,{\rm{S}}({\rm{d}}))$$

where ${{\rm{N}}}_{{\rm{i}}}({\rm{d}})$ is the number of seeded cells and ${\rm{S}}({\rm{d}})={\rm{PE}}\times \exp (\,-\,{\rm{\alpha }}{\rm{d}}-{{\rm{\beta }}{\rm{d}}}^{2})$ the “success” probability for a cell to grow into a colony. Here PE α and β are the model parameters, with PE representing the plating efficiency, i.e. the surviving fraction of unirradiated cells.

The survival fractions after LINAC irradiations were compared through a binomial likelihood ratio test (LRT) and inferences were made using a permutation method^52^.

#### Cell viability

Let *n*_*ij*_ designate the number of viable cells remaining *t*_*i*_ days after radiation exposure to *d*_*j*_ Gy and *n*_*i*0_ the number of viable cells in the control sample at the same time point. We modeled the log ratio $L{R}_{ij}=log\left(\frac{{n}_{ij}}{{n}_{i0}}\right)$ as a bivariate function on time *t*_*i*_ and dose *d*_*j*_ through the regression:

$$L{R}_{ij}={\beta }_{2.5{\rm{Gy}}.{{\rm{\min }}}^{-1}}({t}_{i},\,{d}_{j})+{\chi }_{0.63{\rm{Gy}}.{{\rm{\min }}}^{-1}}\times {\beta }_{0.63{\rm{Gy}}.{{\rm{\min }}}^{-1}vs2.5{\rm{Gy}}.{{\rm{\min }}}^{-1}}({t}_{i},\,{d}_{j}\,)+{\varepsilon }_{ij}$$

where ${\beta }_{2.5{\rm{Gy}}.{{\rm{\min }}}^{-1}}$ and ${\beta }_{0.63{\rm{Gy}}.{{\rm{\min }}}^{-1}vs2.5{\rm{Gy}}.{{\rm{\min }}}^{-1}}$ represent two bivariate penalized B-spline functions, ${\chi }_{0.63{\rm{Gy}}.{{\rm{\min }}}^{-1}}$ a dummy variable indicating cell irradiation under $0.63\,{\rm{Gy}}.{{\rm{\min }}}^{-1}$ dose rates and ${{\rm{\varepsilon }}}_{{\rm{ij}}}$ error terms.

Thus, by considering the $0.63\,{\rm{Gy}}.{{\rm{\min }}}^{-1}$ dose rate irradiation as reference, the comparison in time and dose between the viable cells for the two dose rates is driven by the function ${\beta }_{0.63{\rm{Gy}}.{{\rm{\min }}}^{-1}vs2.5{\rm{Gy}}.{{\rm{\min }}}^{-1}}$:

$$\frac{Cell\,Viability\,0.63\,Gy.mi{n}^{-1}}{Cell\,Viability\,2.5\,Gy.mi{n}^{-1}}=exp({\beta }_{0.63{\rm{Gy}}.{{\rm{\min }}}^{-1}vs2.5{\rm{Gy}}.{{\rm{\min }}}^{-1}}(t,\,d))$$

Computations for this study were carried out using MATLAB Software, version 8.2.0.701 (Mathworks R2013b) and the REFUND package of R software.

#### Cell cycle

The dose rate dependence of the modeling of cell cycle distribution was studied using a compositional data analysis approach. The vectors listing the proportions of cells in the G1, S and G2 phases of the cell cycle are by definition constrained, since their parts sum to one and this perfect multicollinearity renders invalid the usual statistical approaches for unconstrained variables.

Aitchison geometry^53^ provides an adequate treatment of these compositional data by considering ratios of their parts. In the present study, each cycle phase distribution vector [${x}_{{G}_{1}}$, ${x}_{S}$, ${x}_{{G}_{2}}$] was converted into an unconstrained two-component vector [*Alr*_1_1*Alr*_2_] through an additive log-ratio transformation (Alr)

$$Al{r}_{1}=\,\log \left(\frac{{x}_{S}}{{x}_{{G}_{1}}}\right)\,{\rm{and}}\,Al{r}_{2}=\,\log \left(\frac{{x}_{{G}_{2}}}{{x}_{{G}_{1}}}\right)$$

These *Alr* coordinates were then modeled as a bivariate process in dose and time according to their dose-rate exposure:

$$Al{r}_{k}({\delta }_{i},{t}_{j})={a}_{k}({\delta }_{i},{t}_{j})+{R}_{ij}\times {b}_{k}({\delta }_{i},{t}_{j})+{\varepsilon }_{ij}\,{\rm{with}}\,{\rm{k}}=1,2$$

where *a*_*k*_ and *b*_*k*_ are smooth bivariate functions on doses ${{\rm{\delta }}}_{{\rm{i}}}(1\le {\rm{i}}\le {\rm{n}})$ and times ${{\rm{t}}}_{{\rm{j}}}(1\le {\rm{j}}\le {\rm{m}})$. *R*_*ij*_ and ${\varepsilon }_{ij}$ are dose rate indicator variables (zero for 2.5 Gy.min^−1^ and one for 0.63 Gy.min^−1^) and error terms, respectively.

The significant difference of the G1, S and G2 phase distributions between the two dose rates was assessed according to the presence or not of one in the pointwise confidence intervals of the ratios:

$$\frac{{p}_{{G}_{1}}^{0.63\,{\rm{Gy}}.{{\rm{\min }}}^{-1}}({\delta }_{i},{t}_{j})}{{p}_{{G}_{1}}^{2.5\,{\rm{Gy}}.{{\rm{\min }}}^{-1}}({\delta }_{i},{t}_{j})},\,\frac{{p}_{S}^{0.63\,{\rm{Gy}}.{{\rm{\min }}}^{-1}}({\delta }_{i},{t}_{j})}{{p}_{S}^{2.5\,{\rm{Gy}}.{{\rm{\min }}}^{-1}}({\delta }_{i},{t}_{j})}\,{\rm{and}}\,\frac{{p}_{{G}_{2}}^{0.63\,{\rm{Gy}}.{{\rm{\min }}}^{-1}}({\delta }_{i},{t}_{j})}{{p}_{{G}_{2}}^{2.5\,{\rm{Gy}}.{{\rm{\min }}}^{-1}}({\delta }_{i},{t}_{j})}$$

where the letter p designates the fitted probability to be in a given phase cycle computed as:

$$\begin{array}{rcl}{p}_{{G}_{1}}({\delta }_{i},{t}_{j}) & = & \frac{1}{1+\exp (Al{r}_{1}({\delta }_{i},{t}_{j}))+\exp (Al{r}_{2}({\delta }_{i},{t}_{j}))}\\ {p}_{S}({\delta }_{i},{t}_{j}) & = & \frac{Al{r}_{1}({\delta }_{i},{t}_{j})}{1+\exp (Al{r}_{1}({\delta }_{i},{t}_{j}))+\exp (Al{r}_{2}({\delta }_{i},{t}_{j}))}\\ {p}_{{G}_{2}}({\delta }_{i},{t}_{j}) & = & \frac{Al{r}_{2}({\delta }_{i},{t}_{j})}{1+\exp (Al{r}_{1}({\delta }_{i},{t}_{j}))+\exp (Al{r}_{2}({\delta }_{i},{t}_{j}))}\end{array}$$

#### Flow cytometry data analysis

For each radiation dose, the associated 2D scatter points (formed by the forward scatter (FSC) and β-galactosidase staining intensities [FITC]) were summarized by a two-component vector consisting of log-mean intensities in each dimension.

Thus, the dose and dose-rate profile variations of this central tendency was modeled as a flexible spline function in dose:

$$\Delta {C}_{i}^{FSC\,or\,FITC}={\alpha }^{FSCorFITC}({\delta }_{i})+{R}_{i}\times {\beta }^{FSCorFITC}({\delta }_{i})+{\varepsilon }_{i}$$

Where $\Delta {C}_{i}^{FSC\,or\,FITC}$ represents the difference between the exposed (to the dose ${\delta }_{i}$) and the non-exposed mean intensities of FSS or FITC data, ${\alpha }^{FSCorFITC}$ and ${\beta }^{FSCorFITC}$ smooth spline functions, *R*_*i*_ a dummy variable indicating the type of dose rate (0.63 Gy.min^−1^ or 2.5 Gy.min^−1^) and $\varepsilon$ is an error sampled from the zero expectation distribution.

Thus, the function ${\beta }^{FSCorFITC}({\delta }_{i})$ summarizes the rate-dose modulation of the center of the 2D flow cytometry scatterplots.

All the smoothing spline estimations and inferences were performed using the REFUND Package of R software^54^.

#### Gene expression

The measured HUVEC transcriptional profiles for each gene at each time point (after 3, 7 and 21 days) can be viewed as a noisy discretization of a continuous dose-dependent process, denoted by f:

$$-\varDelta {C}_{Ti}=\alpha ({\delta }_{i})+{R}_{i}\times \beta ({\delta }_{i})+{\varepsilon }_{i}$$

Where $-\varDelta {C}_{Ti}$ represents the opposite of the measured $\varDelta {C}_{T}$ at the *i*^*th*^ dose ${\delta }_{i}\,(1\le i\le n)$, *R*_*i*_ is a dummy variable indicating the type of dose rate (0.63 Gy.min^−1^ or 2.5 Gy.min^−1^), $\alpha$ and $\beta$ are smooth functions and $\varepsilon$ is an error sampled from the zero expectation distribution. Thus, the function $\beta (\delta )$ can be viewed as a log-fold change (LFC) of a gene expression profile between the two dose rates for a given dose $\beta (\delta )$.

These LFC profiles, reduced to their significant parts, were then clustered using a two-stage clustering method: after a dimension reduction step using functional principal component analysis (FPCA)^55,56^, clustering on the scores was done using a hierarchical complete-linkage algorithm.

*In vivo*

The ulceration RBE was defined as the ratio of ulceration percentages and the statistical inference about its significance was made using resampling methods^52^.

## Supplementary information

Supplementary Information.
