# Supplementary material for: Variation of 4 MV X-ray dose rate strongly impacts biological response both in vitro and in vivo
Source: Sci Rep. 2020 Apr 27;10:7021. doi: 10.1038/s41598-020-64067-4 (PMC7184727; doi:10.1038/s41598-020-64067-4)
Supplement: Supplementary file 1 — Supplementary Information. [file 41598_2020_64067_MOESM1_ESM.pdf]

Variation of 4 MV X-ray dose rate strongly impacts biological response both *in vitro* and *in vivo*

M Ben Kacem, MA Benadjaoud, M Dos Santos, F Soysouvanh, V Buard, G Tarlet, B Le Guen, A François, O Guipaud, F Milliat and V Paget

Supplementary Information

SF	0.63 $Gy.min^{-1}$ vs 2.5 $Gy.min^{-1}$	95% CI*
0.01	1.11	[1.06;1.16]
0.02	1.14	[1.09;1.19]
0.05	1.21	[1.15;1.27]
0.10	1.21	[1.16;1.27]
0.15	1.20	[1.14;1.27]
0.20	1.19	[1.12;1.27]
0.25	1.18	[1.10;1.27]
0.30	1.17	[1.08;1.27]
0.35	1.16	[1.06;1.28]
0.40	1.16	[1.05;1.28]
0.45	1.15	[1.03;1.28]
0.50	1.15	[1.02;1.29]
0.55	1.14	[1.00;1.29]
0.60	1.14	[0.99;1.29]#
0.65	1.13	[0.98;1.30]#
0.70	1.13	[0.97;1.30]#
0.75	1.12	[0.96;1.30]#
0.80	1.12	[0.95;1.30]#
0.85	1.11	[0.94;1.31]#
0.90	1.11	[0.93;1.31]#

Supplementary Table S1: Clonogenic assay RBE values (Ratio of doses for a given SF).

* 95% CI = 95% confidence interval. # Not statistically significant (CI including 1).

Dose (Gy)	RBE	95% CI*
0.5	1.01	[0.87 ; 1.18]#
1	0.97	[0.78 ; 1.23] #
1.5	0.91	[0.70 ; 1.20] #
2	0.83	[0.62 ; 1.10] #
2.5	0.75	[0.57 ; 0.98]
3	0.68	[0.51 ; 0.88]
3.5	0.62	[0.47 ; 0.79]
4	0.58	[0.44 ; 0.74]
4.5	0.56	[0.43 ; 0.71]
5	0.54	[0.42 ; 0.69]
5.5	0.53	[0.41 ; 0.68]
6	0.53	[0.41 ; 0.68]
6.5	0.53	[0.40 ; 0.67]
7	0.53	[0.40 ; 0.67]
7.5	0.53	[0.40 ; 0.68]
8	0.53	[0.40 ; 0.68]
8.5	0.53	[0.40 ; 0.68]
9	0.53	[0.40 ; 0.69]
9.5	0.53	[0.40 ; 0.69]
10	0.53	[0.40 ; 0.70]
10.5	0.54	[0.40 ; 0.70]
11	0.54	[0.40 ; 0.71]
11.5	0.54	[0.40 ; 0.72]
12	0.55	[0.40 ; 0.72]
12.5	0.55	[0.41 ; 0.72]
13	0.55	[0.41 ; 0.72]
13.5	0.56	[0.41 ; 0.73]
14	0.56	[0.41 ; 0.74]
14.5	0.56	[0.42 ; 0.75]
15	0.57	[0.42 ; 0.75]
15.5	0.57	[0.42 ; 0.75]
16	0.57	[0.42 ; 0.76]
16.5	0.58	[0.42 ; 0.76]
17	0.58	[0.43 ; 0.77]
17.5	0.58	[0.43 ; 0.78]
18	0.59	[0.43 ; 0.79]
18.5	0.59	[0.43 ; 0.79]
19	0.59	[0.43 ; 0.80]
19.5	0.59	[0.43 ; 0.81]
20	0.60	[0.43 ; 0.82]

Supplementary Table S2: Senescence RBE (ratio for a given dose). * 95% CI = 95% confidence interval. # Not statistically significant (CI including 1).

	Estimated RBE	95% CI*
Severe damage	0.5117	[0.4487 ; 0.5834]

Supplementary Table S3: Severe damage RBE (ratio of severe damage percentages for a given dose) * 95% CI = 95% confidence interval. # Not statistically significant (CI including 1).

A.

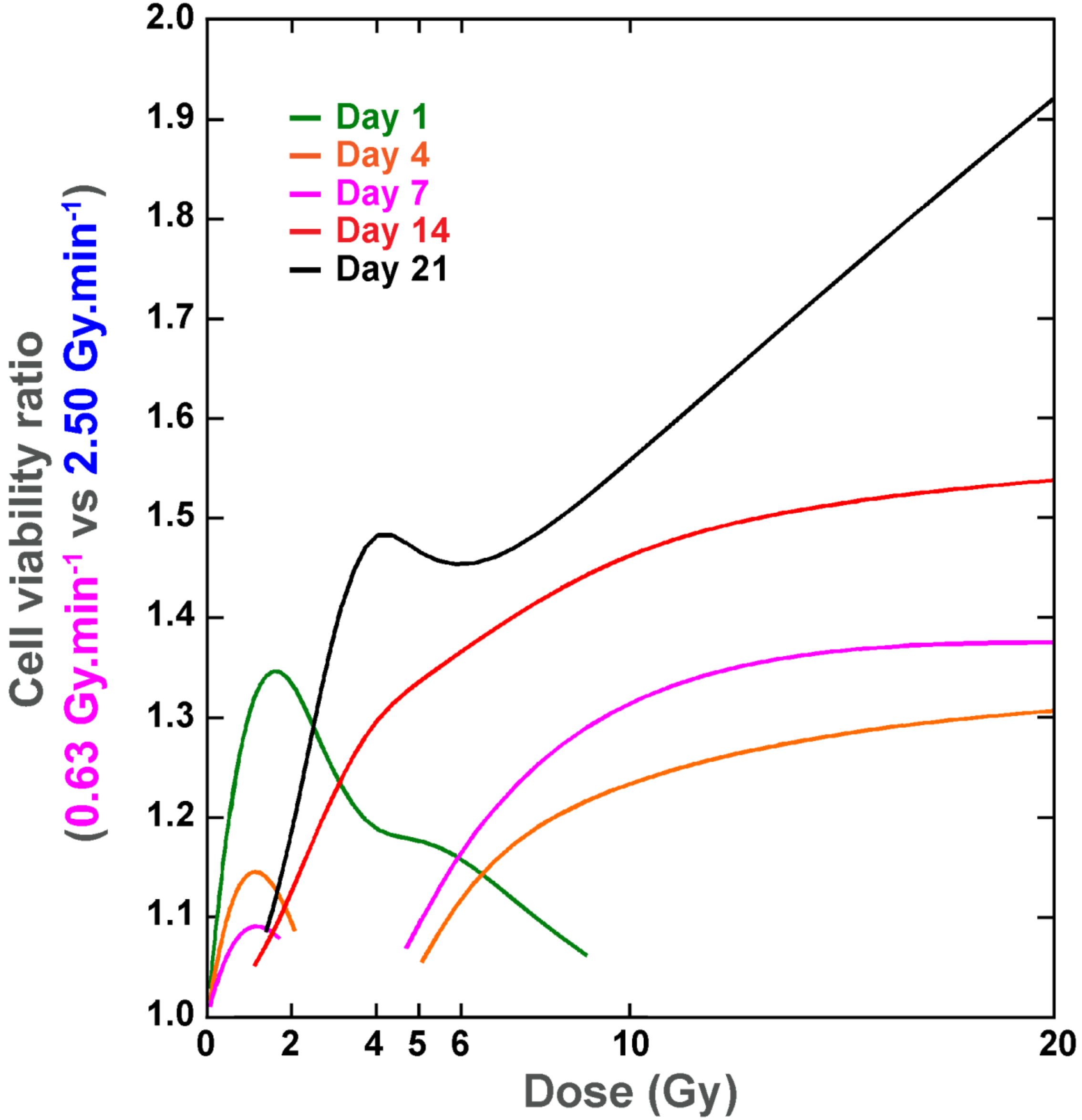

B.

Dose (Gy)	Time (Days)	RBE	95% CI*
2.00	1.00	1.34	[1.23;1.46]
2.00	2.00	1.31	[1.21;1.42]
2.00	3.00	1.22	[1.13;1.31]
2.00	4.00	1.11	[1.03;1.19]
2.00	7.00	1.07	[1.00;1.15]
2.00	14.00	1.11	[1.06;1.18]
2.00	21.00	1.16	[1.09;1.24]
4.00	1.00	1.20	[1.12;1.29]
4.00	2.00	1.13	[1.06;1.20]
4.00	3.00	1.05	[0.99;1.12]#
4.00	4.00	0.97	[0.92;1.03]#
4.00	7.00	1.01	[0.96;1.06]#
4.00	14.00	1.28	[1.22;1.35]
4.00	21.00	1.47	[1.39;1.56]
5.00	1.00	1.18	[1.11;1.26]
5.00	2.00	1.14	[1.08;1.20]
5.00	3.00	1.09	[1.03;1.14]
5.00	4.00	1.03	[0.97;1.08]#
5.00	7.00	1.07	[1.02;1.12]
5.00	14.00	1.33	[1.26;1.39]
5.00	21.00	1.47	[1.40;1.55]
6.00	1.00	1.16	[1.09;1.23]
6.00	2.00	1.16	[1.10;1.23]
6.00	3.00	1.15	[1.09;1.21]
6.00	4.00	1.11	[1.05;1.17]
6.00	7.00	1.16	[1.10;1.21]
6.00	14.00	1.36	[1.30;1.42]
6.00	21.00	1.45	[1.38;1.54]
10.00	1.00	1.05	[0.99;1.11]#
10.00	2.00	1.12	[1.05;1.19]
10.00	3.00	1.18	[1.11;1.25]
10.00	4.00	1.23	[1.16;1.30]
10.00	7.00	1.31	[1.24;1.38]
10.00	14.00	1.46	[1.39;1.52]
10.00	21.00	1.54	[1.45;1.65]
20.00	1.00	0.96	[0.87;1.05]#
20.00	2.00	1.08	[1.00;1.18]
20.00	3.00	1.18	[1.09;1.27]
20.00	4.00	1.31	[1.21;1.41]
20.00	7.00	1.38	[1.28;1.48]
20.00	14.00	1.54	[1.44;1.65]
20.00	21.00	1.92	[1.77;2.09]

Supplementary Figure S1. "RBE" based on cell viability (ratios for a given dose and time). (A) "RBE" representation at day 1 (green curve), day 4 (orange curve), day 7 (purple curve), day 14 (red curve) and day 21 (black curve) as a function of dose (Gy) (B) Numerical values of RBE extracted from experimental data. *95% CI = 95% confidence interval. # Not statistically significant (CI includes 1).

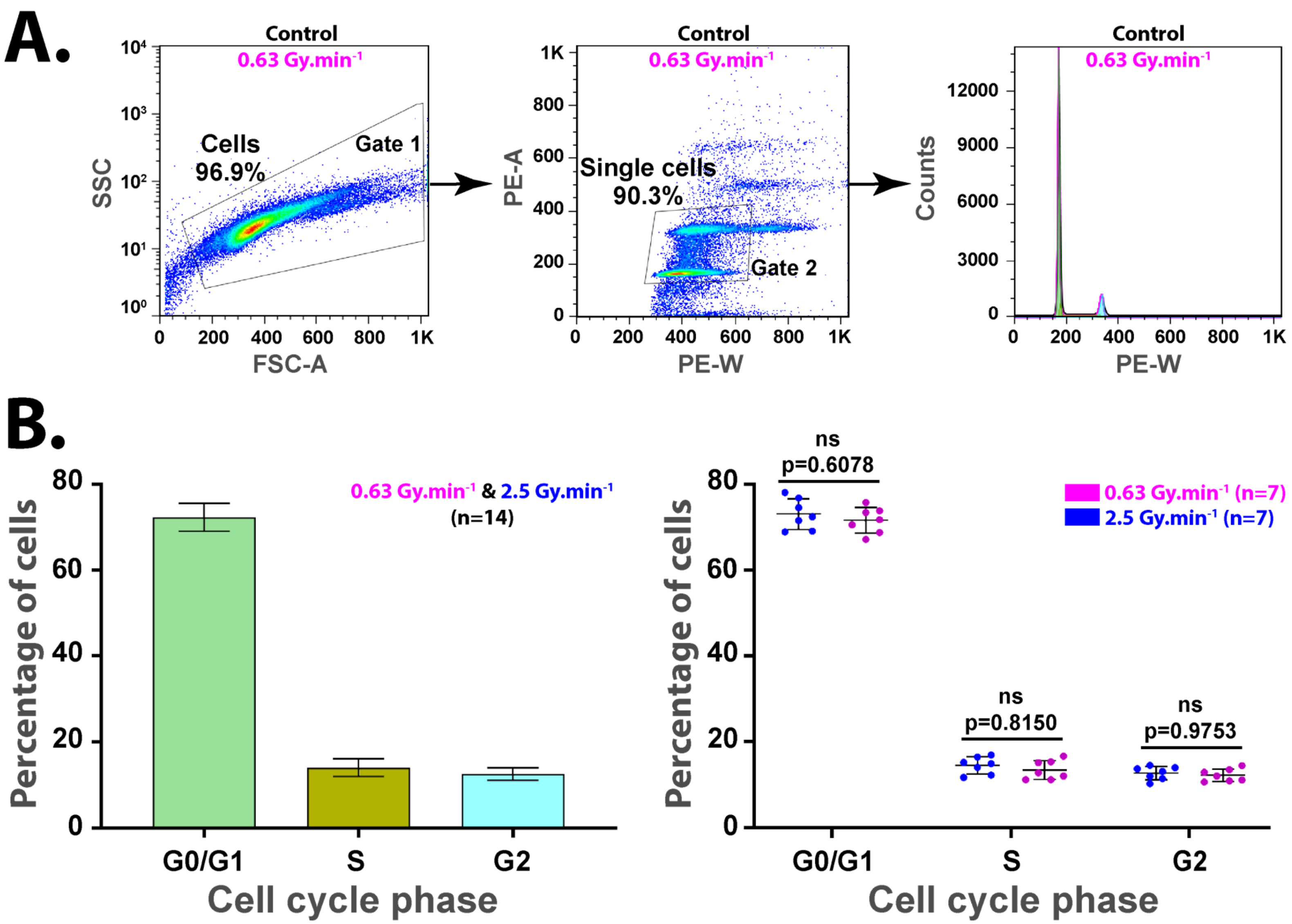

Supplementary Figure S2. Cell cycle analysis performed on controls just before irradiation. (A) Example of one representative cell cycle analysis performed using the FlowJo cell cycle tool on single cells (set up to remove debris first, then by using size (APC-W)/intensity (APC-A) bi-parametric analysis to remove doublets) (B) Mean of cell cycle proportions for controls at T0 (n=14 in total, left panel); n=7 for 0.63 $Gy.min^{-1}$ irradiation (purple plots) plus n=7 for 2.5 $Gy.min^{-1}$ (blue plots) (right panel); 5x104 cells were analyzed per n.

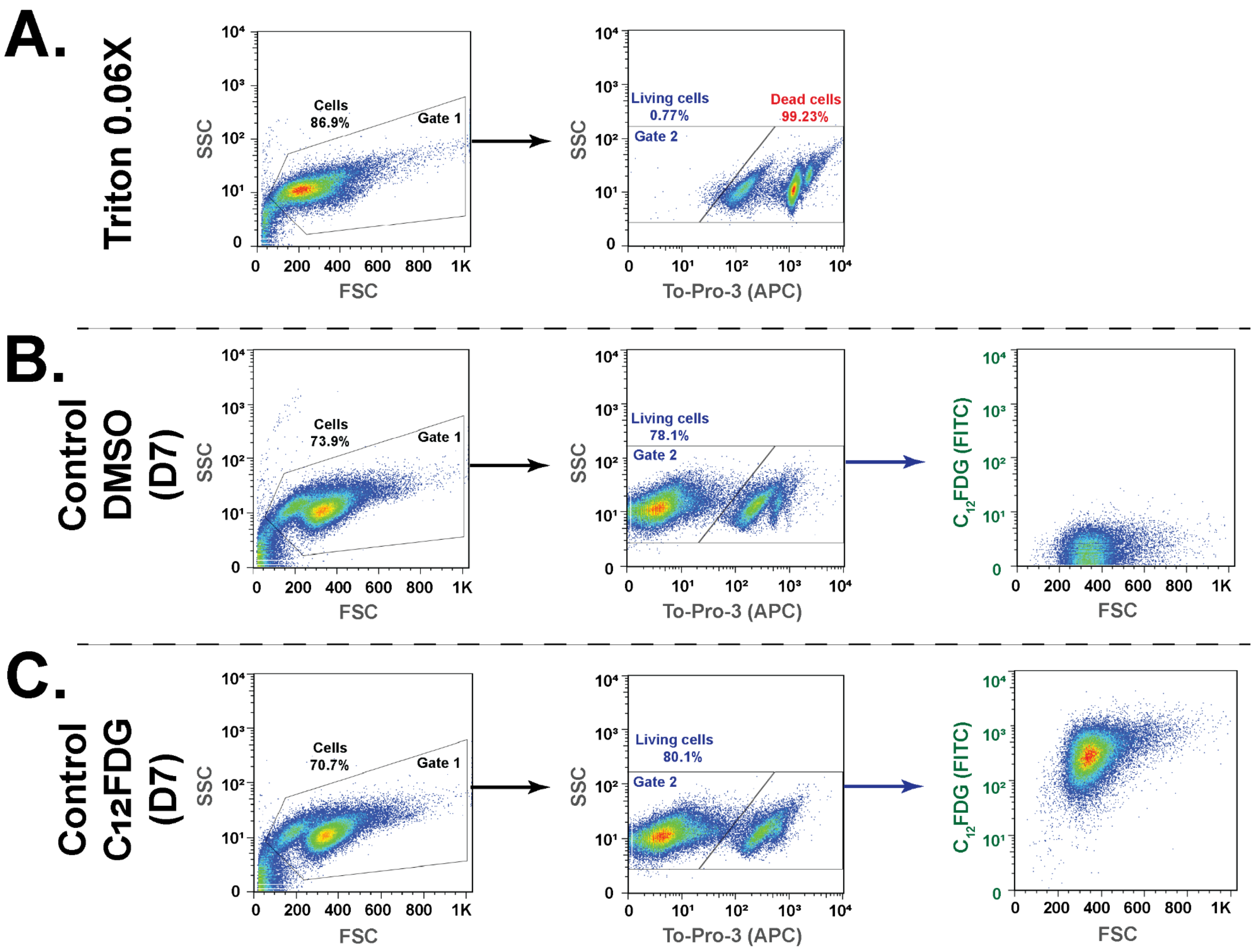

A.
Triton 0.06X
Cells
86.9%
Gate 1
SSC
FSC
Living cells
0.77%
Dead cells
99.23%
Gate 2
To-Pro-3 (APC)
B.
Control
DMSO
(D7)
Cells
73.9%
Gate 1
Living cells
78.1%
Gate 2
C12FDG (FITC)
C.
Control
C12FDG
(D7)
Cells
70.7%
Gate 1
Living cells
80.1%
Gate 2

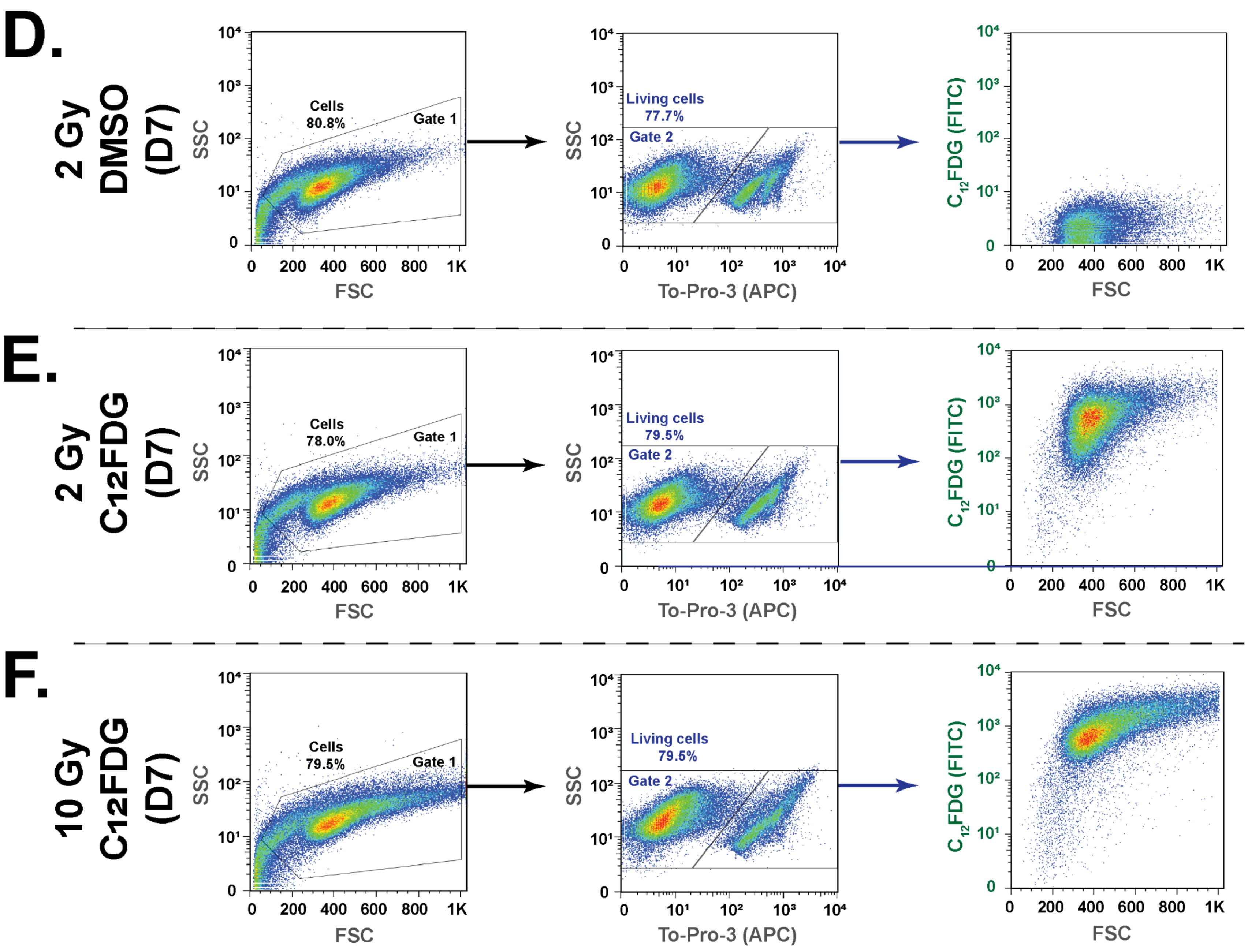

D.
2 Gy DMSO (D7)
E.
2 Gy C12FDG (D7)
F.
10 Gy C12FDG (D7)
SSC
FSC
Cells
80.8%
78.0%
79.5%
Gate 1
To-Pro-3 (APC)
Living cells
77.7%
79.5%
79.5%
Gate 2
C12FDG (FITC)
0
200
400
600
800
1K
10¹
10²
10³
10⁴

G.

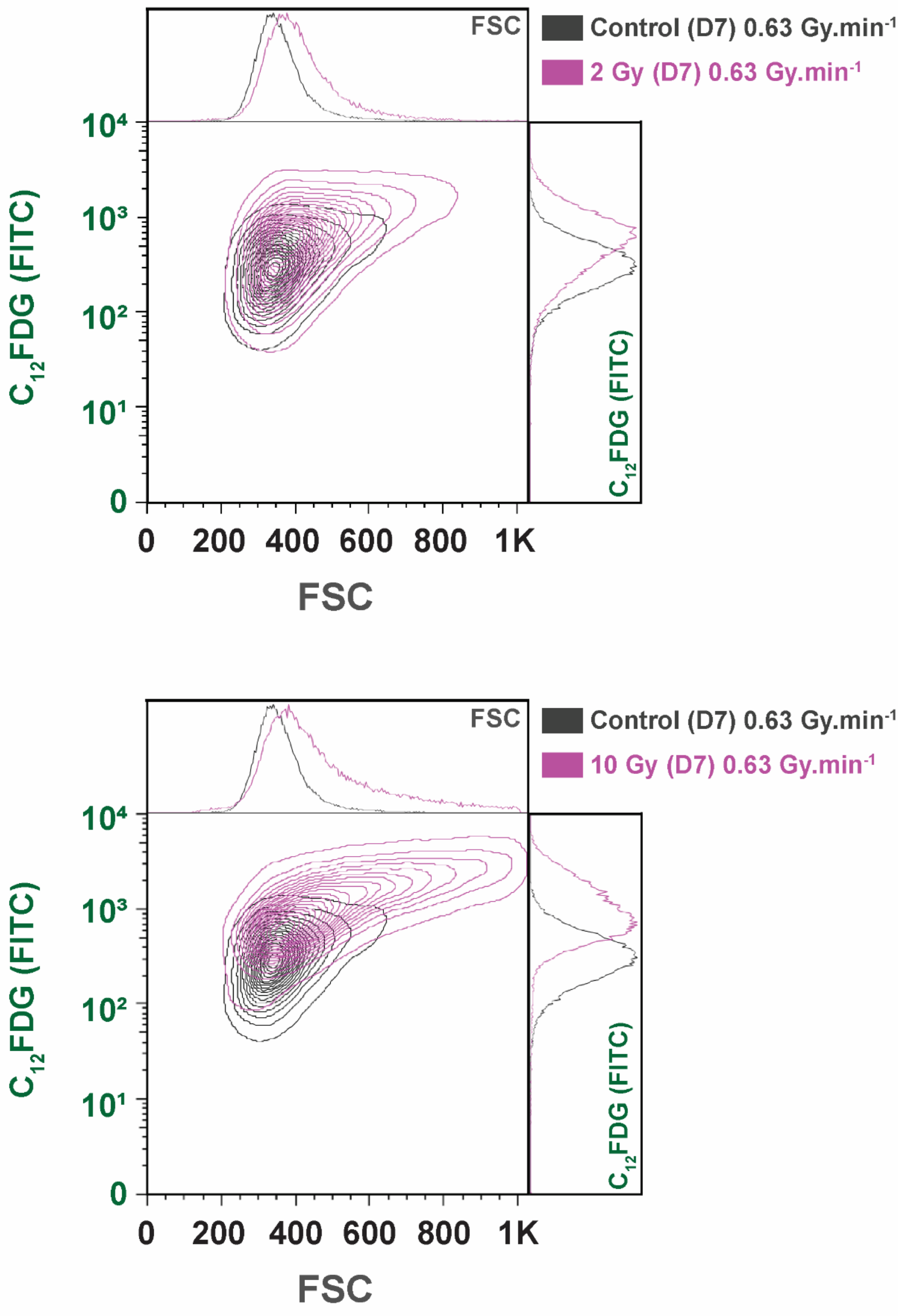

Supplementary Figure S3. Example of analysis set-up for C_{12}FDG experiments for 0.63 Gy.min^{-1} irradiation. (A) First analysis on size (FSC: forward scatter)/granulometry (SSC: side scatter) parameters, to collect cells (gate G1) and to remove fragmented cells and debris. Triton 0.06X final was used as positive control to ensure good detection of living cells (Gate 2) and dead cells. (B) DMSO (vehicle of C_{12}FDG) used as negative control to ensure good detection of C_{12}FDG in controls (C) Detection of C_{12}FDG in controls. (D) DMSO used as negative control to ensure good detection of C_{12}FDG in 2 Gy. (E) Detection of C_{12}FDG in 2 Gy. (F) Detection of C_{12}FDG in 10 Gy. (G) Bi-parametric (size/FITC) overlay of control versus 2 Gy irradiation (upper panel) and control vs 10 Gy irradiation (bottom panel).

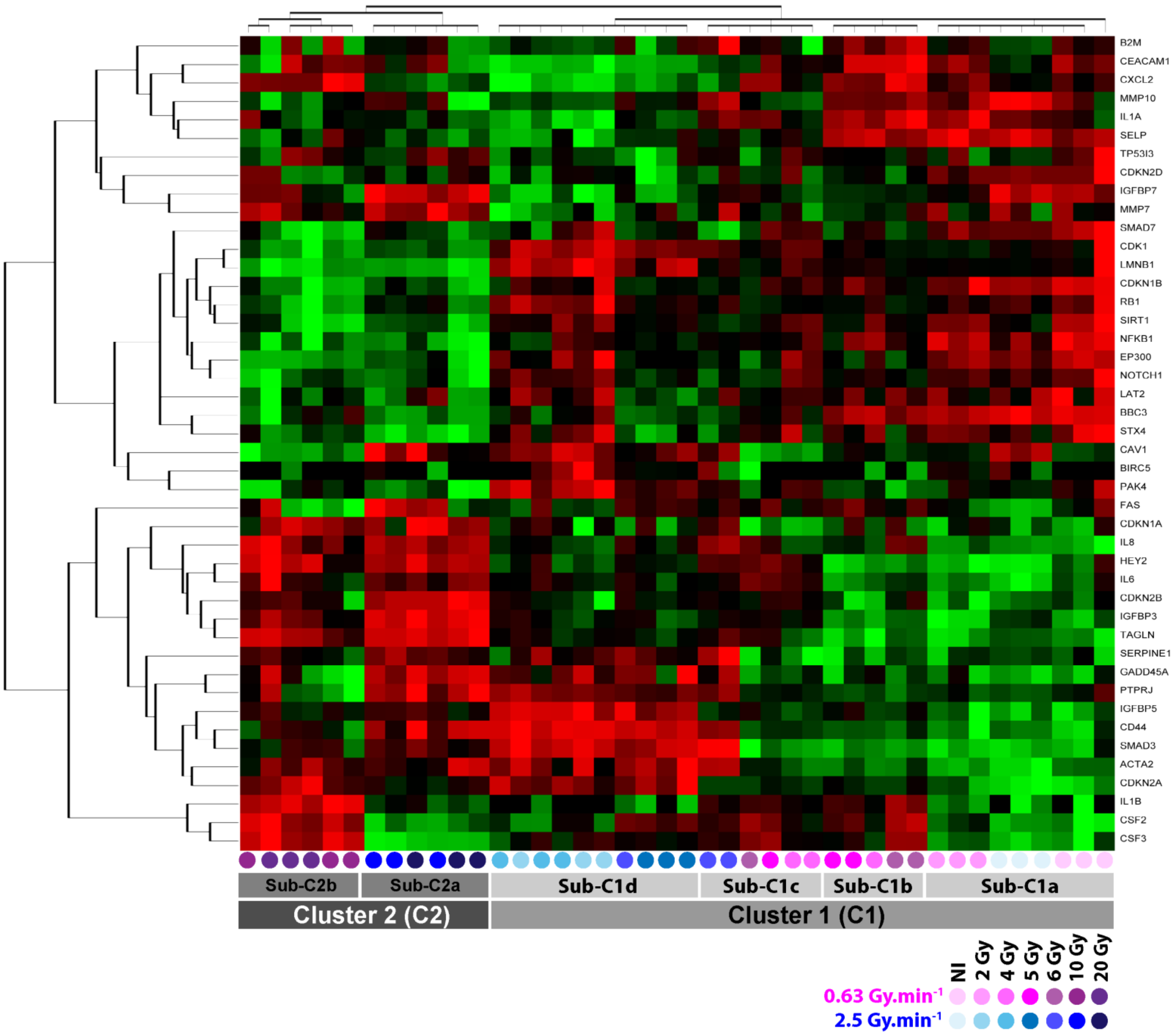

Supplementary Figure S4. RT-qPCR TLDA custom array at D21. Assay heat map illustrating differentially expressed genes among 44 genes measured at day 21 post-irradiation. For one gene considered, red dots represent overexpression and green dots represent downregulation. Corrected p-values using the Benjamini-Hochberg method, $p < 0.05$. ($n = 4$ independent experiments per condition). Purple rounds correspond to 0.63 Gy.min^{-1} irradiation and blue rounds to 2.5 Gy.min^{-1} irradiation, the color gradient indicating the dose.
